# An Investigation of Oxidative Stress and Thiol/Disulphide Homeostasis in Graves’ Disease

**DOI:** 10.3390/medicina55060275

**Published:** 2019-06-14

**Authors:** Veysel Agan, Hakim Celik, Mehmet Ali Eren, Fatma Zehra Agan, Ozcan Erel, Salim Neselioglu, Ismail Koyuncu, Ataman Gonel

**Affiliations:** 1 Department of Physiology, Medical Faculty, Harran University, 63000 Sanliurfa, Turkey; hakimcell@gmail.com; 2 Department of Endocrinology, Medical Faculty, Harran University, 63000 Sanliurfa, Turkey; drmalieren@hotmail.com (M.A.E.); zehracevheri@hotmail.com (F.Z.A.); 3 Department of Clinical Biochemistry, Medical Faculty, Yıldırım Beyazıt University, 06010 Ankara, Turkey; erelozcan@gmail.com (O.E.); sneselioglu@ybu.edu.tr (S.N.); 4 Department of Medicinal Biochemistry, Medical Faculty, Harran University, 63000 Sanliurfa, Turkey; ismailkoyuncu1@gmail.com (I.K.); atamangonel@gmail.com (A.G.)

**Keywords:** Graves’ disease, thiol/disulphide homeostasis, oxidative stress

## Abstract

*Background and objectives*: The aim of this study was to research oxidative stress and thiol/disulphide homeostasis in Graves’ patients. *Materials and Methods*: The study included 33 Graves’ patients (research group) and 35 healthy subjects (control group). Serum oxidative stress and thiol/disulphide homeostasis (a new and automated spectrophotometric method developed by Erel and Neselioglu) parameters were studied and compared between the groups. *Results:* The native and total thiol levels and the native thiol/total thiol ratio were lower in patients with Graves’ disease compared to the control group (*p* < 0.001, *p* < 0.001, and *p* = 0.006, respectively). TOS (total antioxidant status), PC (protein carbonyl), OSI (Oxidative stress index), and disulphide/native thiol and disulphide/total thiol ratios were determined to be higher in the Graves’ disease group than in the control group (*p* < 0.001, *p* = 0.001, *p* = 0.001, *p* = 0.004, and *p* = 0.006, respectively). In the Graves’ disease group, the free triiodothyronine (FT3) and free thyroxine (FT4) levels were significantly positively correlated with impaired thiol/disulphide homeostasis and oxidative stress parameters (*p* < 0.05). *Conclusion*: The results of the current study demonstrated that oxidative stress and thiol/disulphide homeostasis increased towards disulphide formation due to thiol oxidation in Graves’ disease. In addition, a positive correlation of FT3 and FT4 was observed with oxidative stress parameters and impaired thiol/disulphide homeostasis.

## 1. Introduction

Graves’ disease, which is usually associated with goitre and ophthalmopathy, is an autoimmune thyroid disorder characterized by hyperthyroidism (increased thyroid hormone secretion) [1]. Graves’ disease is the most prevalent reason of hyperthyroidism [2].

Oxidative stress is described as a defect in the balance between the generation of reactive oxygen species (ROS) and antioxidant defenses [3]. This increase in metabolic status leads to the formation of free radicals, which may result in bound oxygen toxicity, also known as erythrocyte antioxidant defense system induction [4]. ROS induce oxidation of thiol groups of sulfur, including amino acids and disulphide bonds [5]. Thiol groups include (–SH) and have a significant role in the antioxidant process as they break down ROS and other free radicals [6]. Thiols are known as mercaptan and play a significant role in homeostasis with respect to oxidation and reduction reactions [7]. The disulphide bonds shaped are reversible so can be transformed back into thiol groups and maintain the homeostatic balance between thiols and disulphide bonds [6,7]. The dynamic thiol/disulphide balance has significant functions in antioxidant protection, programmed cell death, cellular transduction mechanisms, cellular enzymatic efficiency, detoxification, and transcription [6]. If the thiol/disulphide balance is weighted towards the disulphide groups, these essential actions are negatively affected and pathologies develop related to functions and organ structure [8]. It has been suggested than a thiol/disulphide imbalance may result in diseases such as cancer, diabetes mellitus, and cardiovascular diseases [9,10]. An automated spectrophotometric method developed by Erel and Neselioglu is currently used to measure the thiol/disulphide balance and allows specific measurements of the thiol and disulphide levels [11].

Many oxidative stress parameters have been studied in Graves’ disease. However, there has been no previous study investigating thiol/disulphide homeostasis in Graves’ disease. Therefore, the aim of this study is to evaluate oxidative stress and thiol/disulphide homeostasis in Graves’ disease and to compare these results with a healthy group.

## 2. Materials and Methods

The Graves’ disease patients included in this study were enrolled from the Endocrinology Clinic, Harran University Faculty of Medicine, Sanliurfa between July 2016–August 2017. Approval for the study was granted by the Ethics Committee of the Medical Faculty of Harran University (10 June 2016; Ethical Code: 74059997.050.01.04/102). Informed consent was obtained from all the study participants. The patients included were aged 18–55 years with no accompanying chronic illness and no drug use, smoking, or alcohol consumption. Physical examinations were performed and then body mass index (BMI) was calculated from the height and weight measurements. The control group was formed of age-matched, healthy individuals with no known chronic disease.

Graves’ disease diagnosis was based on low thyroid stimulating hormone (TSH) and high FT3 and FT4 clinical parameters and was confirmed by scintigraphy. Fasting 5 ml blood samples were taken from 33 Graves’ patients (15 males and 18 females) and 35 healthy control subjects (12 males and 23 females) for a biochemical analysis. The samples were immediately centrifuged for 10 min at 3500 rpm to separate the serum samples, which were then stored in two separate Eppendorf tubes at −80 °C until assay.

The serum TSH, FT3, and FT4 levels were measured using the chemiluminescence method on a Siemens Healthcare Advia Centaur Care (Siemens, Erlangen, Germany) device. The total antioxidant status (TAS) measurements were made using Rel Assay Diagnostic (Gaziantep, Turkey) brand commercial kits on a Thermo Scientific Varioskan (Thermo Fisher Scientific, Vantaa, Finland) microplate reader system. This reaction was measured spectrophotometrically at 660 nm, and the unit was calculated as mmol Trolox Eqv/L. The Rel Assay Diagnostic brand commercial kits were used for TOS, and measurements were read on the Thermo Scientific Varioskan microplate reader system. This reaction was measured spectrophotometrically at 530 nm, and the unit was calculated as μmol H_2_O_2_Eqv./L. The value of the Oxidative Stress Index (OSI) was determined as the Total Oxidant Level/Total Antioxidant Level, with the results shown in Arbitrary Units (AU). An 8-OHdGElabscience brand ELISA commercial kit was used for measurements with a Thermo Scientific Varioskan microplate reader system. Protein Carbonyl (PC) measurements were taken using the Cayman Chemical commercial colorimetric kit and the Thermo Scientific Varioskan microplate reader system. The albumin, thiol, and disulphide parameters were examined in the Department of Biochemistry of Yıldırım Beyazıt University Faculty of Medicine. TAS, total oxidant status (TOS), OSI, 8-hydroxy-2-deoxyguanosine (8-OHdG), and PC parameters were studied in the Harran University Physiology Department Laboratory.

### 2.1. Thiol/Disulphide Homeostasis Parameters

A new and automated method developed by Erel and Neselioglu was used for the serum thiol/disulphide homeostasis parameters. Serum levels of native thiol and total thiol were measured with spectrophotometry utilizing Cobas c501 (Roche Diagnostics, Indianapolis, IN, USA). First, the level of native thiol was measured after the serum had interacted with 5,5-dithiobis-2-nitrobenzoic acid without any procedure, then to measure the total thiol levels, dynamic disulphide bonds in the serum samples were reduced with sodium borohydride (NaBH4) and free functional thiol groups were formed. Formaldehyde was then used to completely remove the unused NaBH4. The total thiol groups, both reduced and native, were measured after reacting with 5,5′-Dithiobis-(2-nitrobenzoic acid) (DTNB). Since the reduction of a disulphide bond forms two thiol groups, the number of dynamic disulphide bonds is calculated as half the difference between the total thiol and native thiol. The ratios of disulphide/native thiol, disulphide/total thiol, and native thiol/total thiol were also calculated [12].

### 2.2. Statistical Analyses

The results of the study were analyzed using Statistical Package for Social Sciences, version 23 software. The test results were shown as mean ± standard deviation values in the Independent Samples Test. A value of *p* < 0.05 was considered statistically significant. For categorical variables, the Pearson Chi square test was applied to intergroup comparisons. Correlations between the variables were evaluated using a Pearson correlation analysis.

## 3. Results

The demographic and laboratory parameters of the Graves’ patients and the control group are presented in Table 1. The study included 33 Graves patients (15 male and 18 females; mean age 31.94 ± 10.28 years) and 35 healthy control subjects (12 male and 23 females; mean age 33.09 ± 10.65 years). There was no significant difference between the Graves patients and the control group with respect to gender, mean age, or BMI. The albumin levels were 4.79 ± 0.36 (g/L) in Graves’ patients and 4.93 ± 0.51 (g/L) in the control group, which was not significant (*p* = 0.192).

The comparisons of thiol/disulphide and other oxidative stress parameters in the Graves and control groups are shown in Table 2. No significant difference was determined between the groups with respect to TAS, DNA damage (8-OHDG), and disulphide values (0.55 ± 0.15 vs. 0.60 ± 0.15 mmol Trolox Eqv/L, *p* = 0.198; 23.66 ± 3.21 vs. 24.52 ± 3.35 ng/mL, *p* = 0.282; and 21.03 ± 9.10 vs. 17.42 ± 6.67, *p* = 0.066, respectively). Although not statistically significant, the disulphide levels were higher in the Graves patients than in the control group.

Native thiol, total thiol, and native thiol/total thiol were found to be significantly lower in the Graves group than in the control group (397.42 ± 51.26 vs. 454.95 ± 57.89 mmol/L, *p* < 0.001; 439.47 ± 42.02 vs. 489.79 ± 58.05 mmol/L, *p* < 0.001; and 90.22 ± 4.56 vs. 92.82 ± 2.77, *p* = 0.006, respectively).

**Figure 1:**
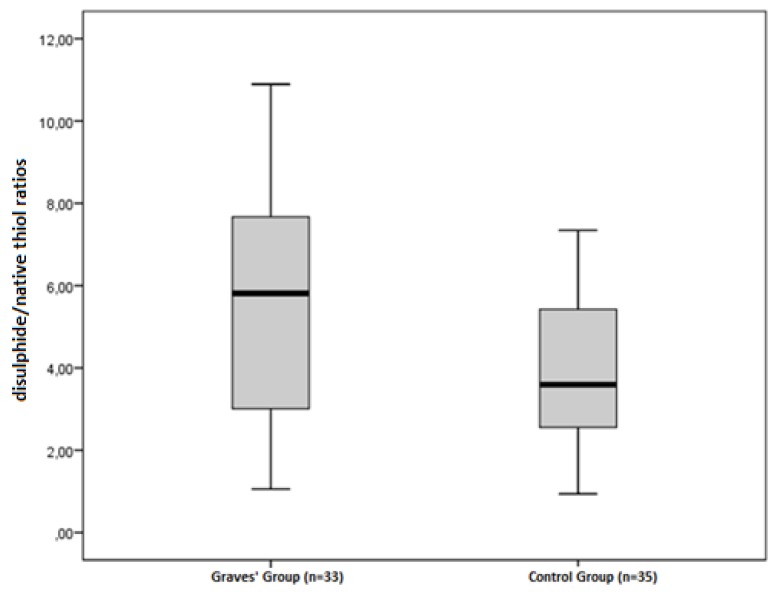
Differences between the disulphide/native thiol ratios in Graves’ patients and control groups.

**Figure 2:**
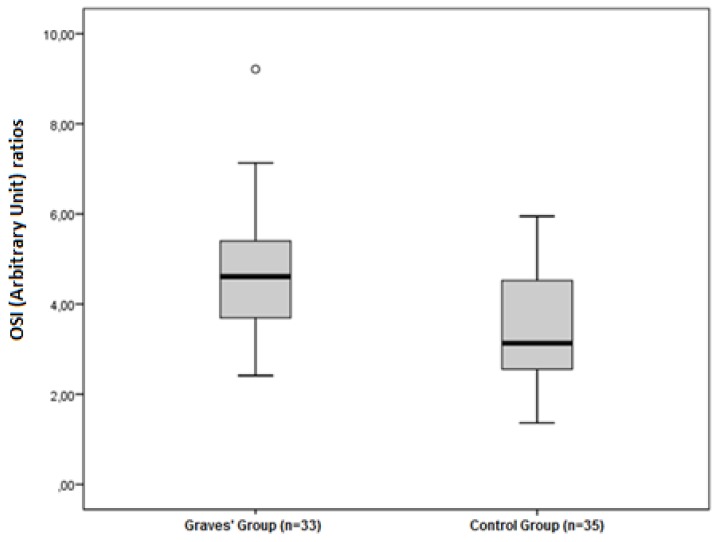
Differences between the OSI (Arbitrary Unit) ratios in Graves’ patients and control groups.

**Figure 3:**
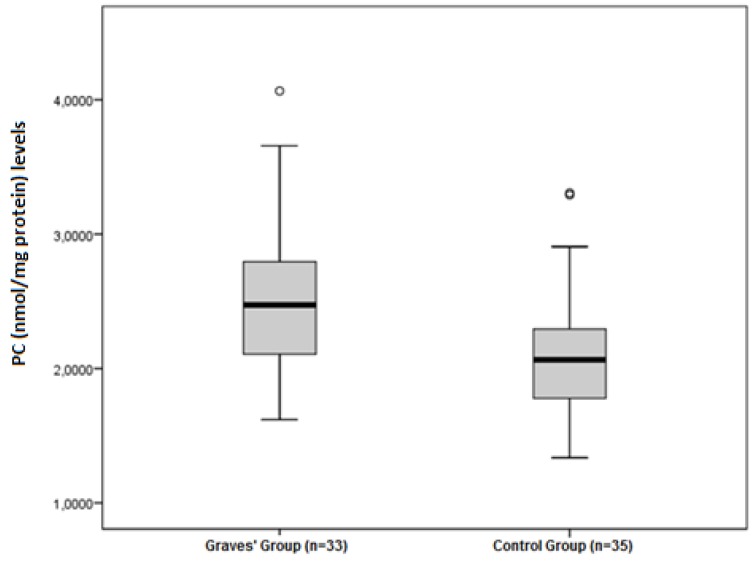
Differences between the PC (nmol/mg protein) levels in Graves’ patients and control groups.

The disulphide/native thiol (Figure 1), the disulphide/total thiol, TOS, OSI (Figure 2), and PC (Figure 3) values were found to be significantly higher in Graves’ patients than in the control group (5.56 ± 2.81 vs. 3.92 ± 1.62, *p* = 0.004; 4.89 ± 2.28 vs. 3.59 ± 1.38, *p* = 0.006; and 2.40 ± 0.34 vs. 1.95 ± 0.44 µmol H_2_O_2_Eqv/L, *p* < 0.001; 4.65 ± 1.46 vs. 3.47 ± 1.27 AU, *p* = 0.001; and 2.52 ± 0.56 vs. 2.09 ± 0.49 nmol/mg protein, *p* = 0.001, respectively).

The correlations of the FT3 and FT4 levels with thiol disulphide and other oxidative stress parameters in Graves’ patients are shown in Table 3.

**Figure 4:**
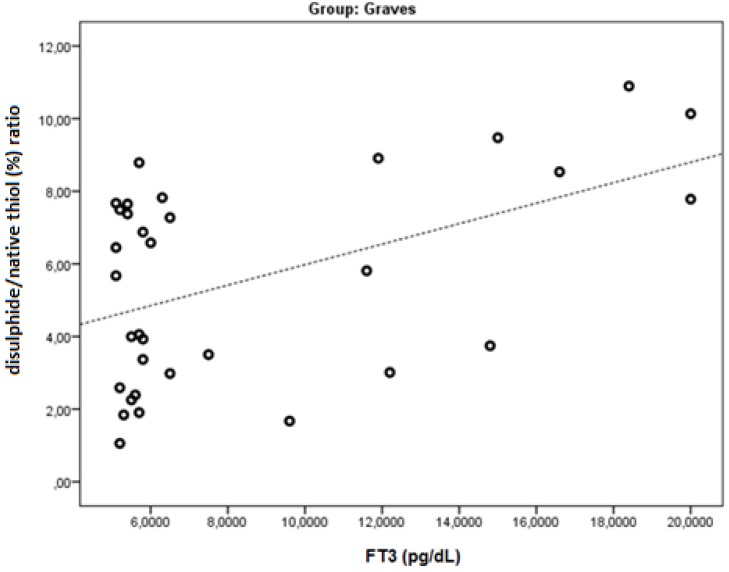
The correlation between the disulphide/native thiol (%) ratio and FT3 levels in Graves’ patients.

**Figure 5:**
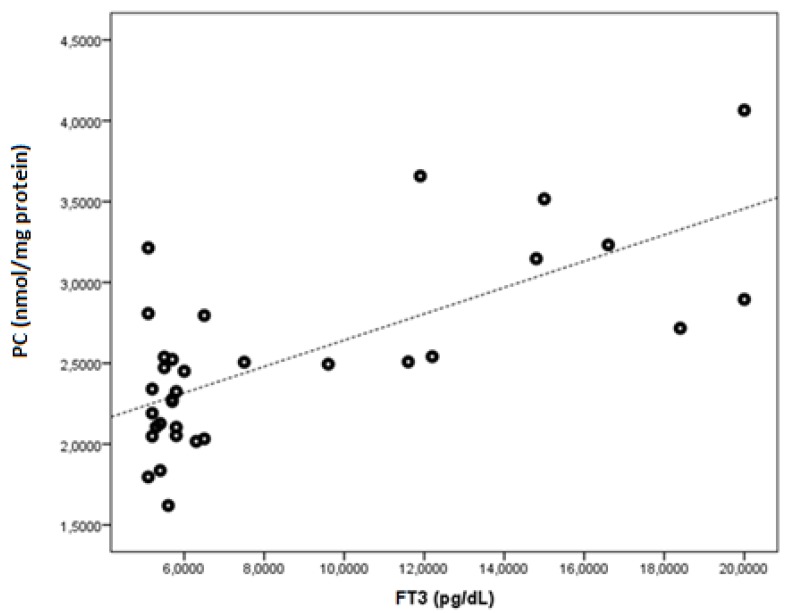
The correlation between the PC (nmol/mg protein) and FT3 levels in Graves’ patients.

**Figure 6:**
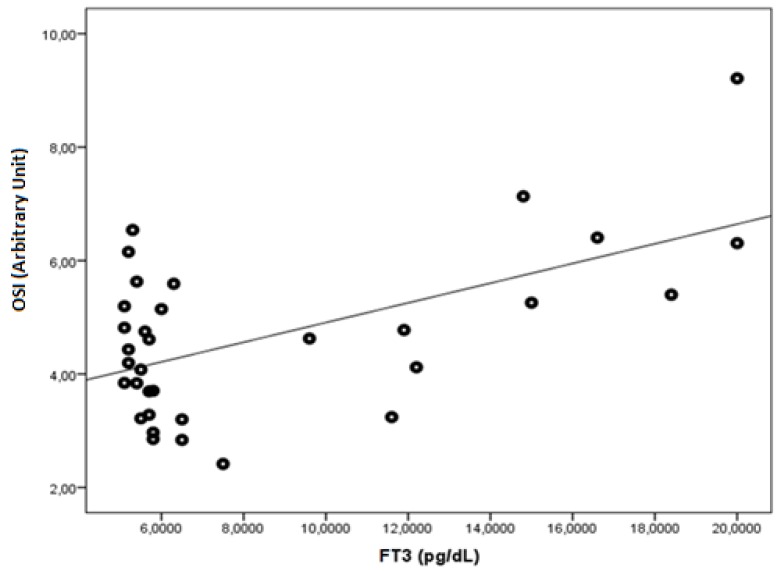
The correlation between the OSI (Arbitrary Unit) and FT3 levels in Graves’ patients.

FT3 was found to be negatively correlated with native thiol, total thiol, and the native thiol/total thiol ratio (r = −0.543, *p* = 0.001; r = −0.505, *p* = 0.003; and r = −0.464, *p* = 0.006, respectively). FT3 was found to be positively correlated with the disulphide, disulphide/native thiol (Figure 4), disulphide/total thiol, PC (Figure 5), TOS, and OSI (Figure 6) values (r = 0.364, *p* =0.037; r = 0.481, *p* = 0.005; r = 0.464, *p* = 0.006; r = 0.7, *p* < 0.001; r = 0.512, *p* = 0.002; and r = 0.569, *p* = 0.001, respectively).

FT4 was found to be negatively correlated with native thiol, total thiol, and the native thiol/total thiol ratio (r = −0.562, *p* = 0.001; r = −0.493, *p* = 0.004; and r = −0.518, *p* = 0.002, respectively). FT4 was found to be positively correlated with the disulphide, disulphide/native thiol, disulphide/total thiol, PC, TOS, and OSI values (r = 0.445, *p* = 0.010; r = 0.525, *p* = 0.002; r = 0.518, *p* = 0.002; r = 0.553, *p* < 0.001; r =0.376, *p* = 0.031; and r = 0.441, *p* = 0.010, respectively).

## 4. Discussion

Thyroid hormones have a regulatory impact on the functions of the cells and tissues in the body. Secretion in low quantities causes the slowing of body functions, and secretion in high quantities causes an acceleration of body functions [13]. Thyroid hormones increase mitochondrial respiration by causing changes in oxygen consumption, mitochondrial oxidative phosphorylation, and the activity and number of certain mitochondrial respiratory chain components [14]. Free radicals are reactive compounds produced naturally by enzymatic and nonenzymatic reactions in the human body and may show positive or negative effects on the body [15]. It has been recently suggested that free radicals have an inducing effect for many diseases [16].

In healthy humans, free radicals are formed in some cellular reactions and they are maintained in balance under physiological conditions through the antioxidant defense systems. When the balance is disrupted, if the free radicals generated are not neutralized, DNA, lipids, and proteins will undergo oxidative damage [17]. Oxidative stress can lead to the development of cancer and result in DNA damage by accelerating mutation and oncogenic transformation [18].

In conditions such as Graves’ disease, hyperthyroidism accelerates the basal metabolism and increases the oxygen consumption required for mitochondrial energy production. This situation increases the production of reactive oxygen radicals. Some tissues try to stabilize with the antioxidant system [19]. In some studies, an increase has been shown in immunological response in the presence of oxidative stress, and this is thought to be related to Graves’ disease [20].

In this study, measurements were taken of TAS, TOS, 8-OHDG, PC values, and OSI and thiol/disulphide ratios with Graves’ disease. Differences and associations were then examined by comparison with the healthy control group. The TOS value and OSI ratio were determined to be significantly higher in the Graves’ disease patients than in the control group (*p* < 0.05). In previous studies, oxidative stress has been seen to increase in Graves’ disease, showing that increased oxidative stress is effective in Graves’ disease. Marcocci et al. [21] reported that oxidative stress was high in Graves’ disease. Aslan et al. [22] and Erdemar et al. [23] stated that oxidative stress was high in hyperthyroidism.

Oxidative stress characterizes not only Graves´ disease but also autoimmune thyroid diseases in general, like Hashimoto’s thyroiditis, even in euthyroid subjects. It was determined that antioxidants decrease and oxidants increase in thyroid diseases such as euthyroid Hashimato (HT). As a result, the oxidative/antioxidative balance is shifted toward the oxidative side. Also, another study IL-37 is upregulated in HT and may exert a protective role by counteracting oxidative stress and inflammation [24,25].

At the same time, the rates of native thiols, total thiols, disulphides and native thiols/total thiols, disulphide/total thiols, and disulphide/native thiols were also examined in this study to evaluate oxidative stress on Graves’ patients. No statistically significant difference was determined between the patient and control groups in terms of disulphide value although the disulphide value of the Graves’ patients was higher than that of the control group. However, compared to the control group, the ratios of native thiols, total thiols, and native thiols/total thiols were found to be statistically significantly lower in the Graves’ patients (*p* < 0.05), and the ratios of disulphide/native thiol and disulphide/total thiol were found to be significantly higher (*p* < 0.05). Elmas et al. [26] said that, in obese children disulphide, the disulphide/native thiol and disulphide/total thiol ratios increased compared to the control group and that the thiol, total thiol, and thiol/total thiol ratios decreased.

In the Graves’ patients of the current study, there was a negative correlation between the ratio of native thiols, total thiols, and native thiols/total thiols and the FT3 and FT4 levels. There was a positive correlation between disulphide, PC, TOS levels and disulphide/native thiol, disulphide/total thiol, and OSI ratios and the FT3 and FT4 levels. This data clearly shows that there is a relationship between the thiol/disulphide balance and the FT3 and FT4 levels.

Thiols are sulfur analogues of alcohols [8]. Disulphides are structures containing adjacent double sulfur atoms. The thiol/disulphide balance plays an important role in antioxidant reactions, detoxification, enzyme activity, apoptosis, transcription, and cellular signal transduction mechanisms.

Otherwise, thiols, which are the main components of intracellular and extracellular damage protection mechanisms, activate the antioxidant properties in the presence of oxidative stress and protect the cells against free oxygen radicals. As a result of the oxidative stress that develops in Graves’ disease, free radicals increase and the thiols available to protect the cells become active and show antioxidant properties. This was observed to lead to a reduction in the amount of thiol and an increase in the amount of disulphide present in the reaction as a result of free radicals.

An abnormal thiol/disulphide balance plays a role in the pathogenesis of various diseases such as cardiovascular diseases, diabetes mellitus, cancer, chronic renal failure, and liver diseases [27]. Plasma protein thiol groups are sensitive to oxidative damage and have been shown to reduce oxidative damage in diseases such as coronary artery disease, rheumatoid arthritis, and diabetes mellitus [28,29].

In addition, some neurodegenerative diseases (such as Alzheimer’s, Parkinson’s, and multiple sclerosis) have proven to have abnormal thiol/disulphide homeostasis [7,11,30].

In the current study, the protein carbonyl level was significantly higher in Graves patients than in the control group (*p* < 0.05). Similarly, Cakatay et al. [31] found that plasma carbonyl levels of hyperthyroid patients were significantly higher than those of the control group. In another study, Venditti et al. [32] found significant increases in protein carbonyl levels in the heart and liver of hyperthyroid rats. This shows that protein damage in Graves’ patients is significantly increased.

The release of sulfur-containing amino acids may be a factor affecting this thiol balance. Interference in the concentration of these amino acids can be used as a method to correct the thiol balance. To the best of our knowledge, there has been no previous research in the literature about the oxidative stress and thiol/disulphide balance in Graves’ patients. Therefore, this study is of value as the first to investigate the thiol/disulphide homeostasis in Graves’ patients.

The limitation of this study is that the variability of oxidant and antioxidant parameters in the treatment process has not been evaluated. The availability of these parameters can be investigated in evaluating the effectiveness of treatment.

## 5. Conclusions

In conclusion, TOS, protein carbonyl, disulphide levels, and OSI ratio were significantly increased and thiol levels were decreased in Graves patients compared to the control group. These changes in the TSH, FT3, and FT4 levels triggered free radical formation and caused the OSI, TOS, protein carbonyl, and disulphide values to rise. The reduction in thiol groups, a natural antioxidant that protects the body from oxidative stress, is a major sign of oxidative damage. The low grade of TSH suggests that the elevated FT3 and FT4 levels cause an alteration and peroxidation of the tissue in the antioxidant defence system.

## Figures and Tables

**Table 1:** Data and some laboratory parameters of the Graves’ patients and the control group.

Data	Control (*n* = 35)	Graves (*n* = 33)	*p*-Values
Age (Years)	33.09 ± 10.65	31.94 ± 10.28	=0.653
Gender (male), n (%)	12 (34.3)	15 (45.5)	=0.885
BMI (kg/m2)	24.16 ± 1.65	24.76 ± 2.39	=0.232
Albumin (g/L)	4.93 ± 0.51	4.79 ± 0.36	=0.192
TSH (µU/mL)	1.94 ± 1.04	0.03 ± 0.07	<0.001
FT3 (pg/dL)	3.06 ± 0.33	8.51 ± 4.79	<0.001
FT4 (ng/dL)	1.01 ± 0.13	2.61 ± 1.33	<0.001

BMI, body mass index; TSH, thyrotrophin stimulating hormone; FT3, free triiodothyronine; FT4, free thyroxine.

**Table 2:** A comparison of thiol/disulphide and other oxidative stress parameters in Graves’ patients and the control group.

Data	Control (*n* = 35)	Graves (*n* = 33)	*p*-Values
Native thiol (mmol/L)	454.95 ± 57.89	397.42 ± 51.26	<0.001
Total thiol (mmol/L)	489.79 ± 58.05	439.47 ± 42.02	<0.001
Disulphide (mmol/L)	17.42 ± 6.67	21.03 ± 9.10	0.066
Disulphide/Native thiol (%)	3.92 ± 1.62	5.56 ± 2.81	0.004
Disulphide/Total thiol (%)	3.59 ± 1.38	4.89 ± 2.28	0.006
Native thiol/Total thiol (%)	92.82 ± 2.77	90.22 ± 4.56	0.006
TAS (mmol TroloxEqv/L)	0.60 ± 0.15	0.55 ± 0.15	0,198
TOS (µmol H2O2 Eqv/L)	1.95 ± 0.44	2.40 ± 0.34	<0.001
OSI (Arbitrary Unite)	3.47 ± 1.27	4.65 ± 1.46	0.001
PC (nmol/mg protein)	2.09 ± 0.49	2.52 ± 0.56	0.001
8-OHdG (ng/mL)	24.52 ± 3.35	23.66 ± 3.21	0.282

TAS, total antioxidant status; TOS, total oxidant status; OSI, oxidative stress index; PC, protein carbonyl; 8-OHdG, 8-Hydroxy-2-deoxyguanosine.

**Table 3:** Correlation analyses of FT3 and FT4 with thiol disulphide homeostasis and the oxidative stress parameters in Graves’ disease.

		Native Thiol	Total Thiol	Disulphide	Disulphide/Native Thiol	Disulphide/Total Thiol	Native Thiol/Total Thiol	PC	8OHdG	TAS	TOS	OSI
FT3	r	−0.543	−0.505	0.364	0.481	0.464	−0.464	0.700	0.267	−0.286	0.512	0.569
	*p*	0.001	0.003	0.037	0.005	0.006	0.006	<0.001	0.134	0.106	0.002	0.001
FT4	r	−0.562	−0.493	0.445	0.525	0.518	−0.518	0.553	0.217	−0.201	0.376	0.441
	*p*	0.001	0.004	0.010	0.002	0.002	0.002	0.001	0.225	0.262	0.031	0.010

FT3, free triiodothyronine; FT4, free thyroxin; TAS, total antioxidant status; TOS, total oxidant status; OSI, oxidative stress index; PC, protein carbonyl; 8-OHdG, 8-Hydroxy-2-deoxyguanosine.
